# Combined associations of visceral adipose tissue and adherence to a Mediterranean lifestyle with T2D and diabetic microvascular complications among individuals with prediabetes

**DOI:** 10.1186/s12933-024-02284-1

**Published:** 2024-06-12

**Authors:** Hao-Wen Chen, Kuan Liu, Bi-Fei Cao, Qi Zhong, Rui Zhou, Liang-Hua Li, Shi-Ao Wang, Yan-Fei Wei, Hua-Min Liu, Xian-Bo Wu

**Affiliations:** 1 Department of Epidemiology, School of Public Health, Guangdong Provincial Key Laboratory of Tropical Disease Research, Southern Medical University, No. 1063-No. 1023, Shatai South Road, Baiyun District, Guangzhou, 510515 China; 2 Public Health Division, Hospital of Zhongluotan Town, Baiyun District, Guangzhou, 510515 China; 3 Department of Anaesthesiology, Nanfang Hospital, Southern Medical University, Guangzhou, 510515 China

## Abstract

**Background:**

It’s unclear if excess visceral adipose tissue (VAT) mass in individuals with prediabetes can be countered by adherence to a Mediterranean lifestyle (MEDLIFE). We aimed to examine VAT mass, MEDLIFE adherence, and their impact on type 2 diabetes (T2D) and diabetic microvascular complications (DMC) in individuals with prediabetes.

**Methods:**

11,267 individuals with prediabetes from the UK Biobank cohort were included. VAT mass was predicted using a non-linear model, and adherence to the MEDLIFE was evaluated using the 25-item MEDLIFE index, encompassing categories such as “Mediterranean food consumption,” “Mediterranean dietary habits,” and “Physical activity, rest, social habits, and conviviality.” Both VAT and MEDLIFE were categorized into quartiles, resulting in 16 combinations. Incident cases of T2D and related DMC were identified through clinical records. Cox proportional-hazards regression models were employed to examine associations, adjusting for potential confounding factors.

**Results:**

Over a median follow-up of 13.77 years, we observed 1408 incident cases of T2D and 714 cases of any DMC. High adherence to the MEDLIFE, compared to the lowest quartile, reduced a 16% risk of incident T2D (HR: 0.84, 95% CI: 0.71–0.98) and 31% for incident DMC (0.69, 0.56–0.86). Conversely, compared to the lowest quartile of VAT, the highest quartile increased the risk of T2D (5.95, 4.72–7.49) and incident any DMC (1.79, 1.36–2.35). We observed an inverse dose-response relationship between MEDLIFE and T2D/DMC, and a dose-response relationship between VAT and all outcomes (*P* for trend < 0.05). Restricted cubic spline analysis confirmed a nearly linear dose-response pattern across all associations. Compared to individuals with the lowest MEDLIFE quartile and highest VAT quartile, those with the lowest T2D risk had the lowest VAT and highest MEDLIFE (0.12, 0.08–0.19). High MEDLIFE was linked to reduced T2D risk across all VAT categories, except in those with the highest VAT quartile. Similar trends were seen for DMC.

**Conclusion:**

High adherence to MEDLIFE reduced T2D and MDC risk in individuals with prediabetes, while high VAT mass increases it, but MEDLIFE adherence may offset VAT’s risk partly. The Mediterranean lifestyle’s adaptability to diverse populations suggests promise for preventing T2D.

**Supplementary Information:**

The online version contains supplementary material available at 10.1186/s12933-024-02284-1.

## Introduction

As of 2021, the global prevalence of prediabetes stands at 9.1% (464 million), with a projected increase to 10.0% (638 million) by the year 2045 [1]. Prediabetes is an intermediate metabolic state between normoglycemia and diabetes, with primary complications leading to diabetic microvascular complications (DMC) such as retinopathy, neuropathy, and nephropathy [2–4]. Considering the substantial public health impact attributed to diabetes, the implementation of early intervention strategies for individuals with prediabetes holds paramount practical significance [2, 5].

Lifestyle intervention, emphasizing a healthy diet and regular exercise, is advocated in diabetes care [6, 7]. While lifestyle interventions for individuals with prediabetes have demonstrated efficacy in mitigating the risk of developing diabetes [2], controversy exists regarding its impact on DMC [8, 9]. Additionally, comprehensive research on a holistic lifestyle approach for mitigating complications in prediabetes is lacking [10]. The Mediterranean lifestyle (MEDLIFE), prevalent in the Mediterranean basin, involves a plant-based diet, moderate consumption of fish and poultry and limited intake of red meat and processed foods, and additional practices such as adequate sleep and heightened social engagement, demonstrates a risk-reducing effect on various diseases, extending beyond Mediterranean regions [11, 12]. While prior studies focused on the Mediterranean dietary pattern in the context of T2D and DMC [13], limited attention has been given to other lifestyle components [14, 15]. Importantly, the impact of MEDLIFE on T2D and DMC in individuals with prediabetes remains unknown.

Obesity is acknowledged as a significant risk factor for T2D and associated complications [16]. While BMI has traditionally been used for obesity classification, its limitations in accurately assessing regional fat distribution may lead to contentious findings [17, 18]. Recent research underscores the pivotal role of visceral adipose tissue (VAT) accumulation, as opposed to subcutaneous adipose tissue (SAT), in precipitating metabolic diseases [17]. Current studies predominantly use indicators like the visceral adiposity index (VAI) and lipid accumulation products (LAP) to assess visceral fat function and its associations with T2D and cardiovascular disease [19–21]. However, there is a limited exploration of the association between VAT mass and these endpoints.

Contemporary recommendations underscore the importance of a healthy diet and maintaining normal body weight to mitigate T2D and cardiovascular diseases [6, 17]. However, uncertainties persist about whether elevated VAT mass in individuals with prediabetes can be mitigated by adherence to a MEDLIFE, and if reduced adherence to a MEDLIFE can be compensated for by a decrease in VAT mass. Our aim was to assess the associations of MEDLIFE and VAT mass with subsequent complications in individuals with prediabetes. We also investigated the combined effects of adherence to MEDLIFE and VAT on T2D and DMC in individuals with prediabetes, using low MEDLIFE adherence and high VAT as the reference.

## Method

### Study participants

**Fig. 1:**
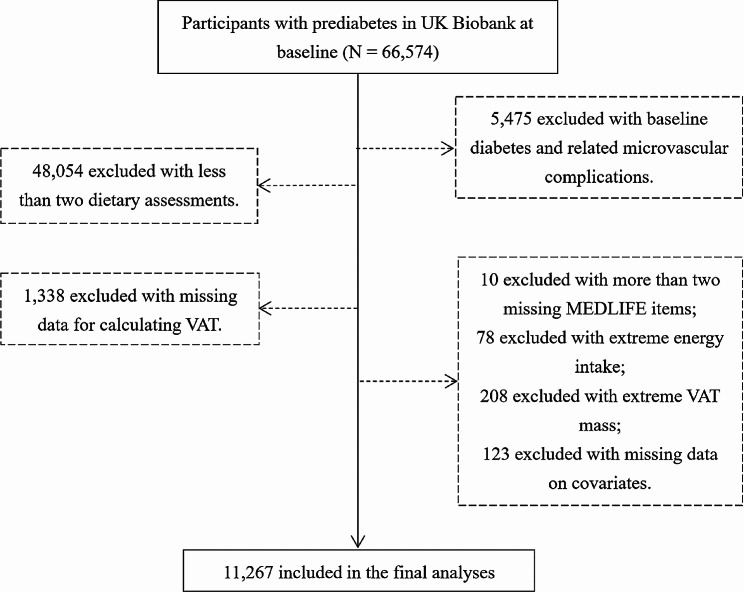
The flowchart for this study

Between 2006 and 2010, the UK Biobank (UKB) enrolled over half a million participants aged 37 to 73 from 22 assessment centres across the United Kingdom. The diverse cohort represented various genetic backgrounds, socioeconomic statuses, and lifestyles. Extensive data, including lifestyle information, physical measurements, biological samples, and health-related outcomes, were collected. (Reference: 11/NW/03820), and all participants provided written informed consent. Participants with prediabetes were identified based on the presence of impaired fasting glucose and/or hemoglobin A1c levels falling within the range of 5.7–6.4% (39–47 mmol/mol), following the 2021 diagnostic criteria outlined in the American Diabetes Association guideline [22]. In our study, 66,574 participants with prediabetes were included (Fig. 1).

### Assessment of mediterranean lifestyle factors

In our study, the adaptation of the MEDLIFE Index for implementation in the UK Biobank study was guided by insights from previously reported studies [12, 23–25]. Specifically, the assessment of the Mediterranean lifestyle diet at baseline (2009–2012) involved up to five web-based 24-hour dietary assessments (Oxford WebQ) [26]. Baseline self-reported data on physical activity, sedentary activities, rest routines, and social habits were gathered at assessment centers between 2006 and 2010. The modified MEDLIFE index utilized in our analysis comprised 25 items instead of 29 (Table S1) due to the unavailability of information on the consumption of olive oil, sofrito (a traditional sauce with olive oil, tomato, and garlic), nibbling outside meals, and eating in company in the UK Biobank dataset. To specify, the MEDLIFE Index consists of three blocks [27]: (a) “Mediterranean food consumption,” encompassing 12 items related to food intake (i.e., sweets, red meat, processed meat, eggs, legumes, fish, potatoes, low-fat dairy products, nuts, fruit, and vegetables); (b) “Mediterranean dietary habits,” encompassing 7 items addressing habits and practices around meals (i.e., adequate wine consumption, low salt consumption, adequate fiber intake, limited snacks intake, healthy beverages consumption, limiting sugar-sweetened beverages); (c) “Physical activity, rest, social habits, and conviviality,” encompassing 6 items focusing on physical activity and collective activities (i.e., physical activity, regular naps, adequate sleep, limited sedentary activities, participating in collective sports, and socializing with friends) (Table S1). Each item is scored as 1 point for adherence or 0 for non-adherence, resulting in a total score ranging from 0 to 25, where higher values indicate greater adherence to the Mediterranean lifestyle.

In our analyses, we included only participants who completed at least two diet assessments, and average scores were incorporated for analysis.

### Assessment of VAT mass

We employed sex-stratified, non-linear prediction models for visceral adipose tissue (VAT) mass using data from the UK Biobank cohort by Karlsson et al. [28]. The models demonstrated high predictive accuracy with R^2^ values of 0.76 (0.74–0.78) for women and 0.77 (0.75–0.78) for men between estimated VAT values and those determined by dual-energy X-ray absorptiometry (DEXA). The minimal mean bias observed, 0.04 g in women and 0.54 g in men, indicates a negligible disparity between estimated VAT values and those determined by DEXA [28]. The formulas for estimating VAT are delineated as follows:

For Males: − 17.02 × age + 8.335 × wst + 10.34 × hip − 50.71 × hgt + 71.21 × wgt + 106.4 × lrm − 35.20 × rrm + 160.5 × llg − 50.25 × rlg − 85.24 × wlb + 0.3914 × age × wgt + 0.3042 × wst × wgt − 0.5459 × hip × wgt − 0.7007 × lrm × hgt + 0.2020 × rrm × hgt − 1.006 × llg × hgt + 0.2949 × rlg × hgt + 0.5900 × wlb × hgt + 2.286. 

For Females: −5.531 × age + 34.84 × hmp − 24.67 × wst + 26.24 × hip − 13.83 × hgt + 26.30 × wgt + 83.11 × lrm − 1.599 × rrm + 89.01 × llg + 24.24 × rlg − 89.59 × wlb + 0.2111 × age × wgt + 0.7424 × wst × wgt − 0.6694 × hip × wgt − 0.5454 × lrm × hgt + 0.005128 × rrm × hgt − 0.5817 × llg × hgt − 0.1415 × rlg × hgt + 0.5903 × wlb × hgt − 281.6.

(hmp, menopausal status; wst, waist circumference; hip, hip circumference; hgt, height; wgt, weight; lrm, impedance of the left arm; rrm, impedance of the right arm; llg, impedance of the left leg; rlg, impedance of the right leg; wlb, impedance of the whole body).

### Definition of outcome

Participants in this longitudinal study were prospectively monitored from the point of recruitment until the occurrence of mortality, loss to follow-up, or until July 11, 2023. Outcomes of this study included T2D and any DMC (including diabetic retinopathy, diabetic neuropathy, and diabetic kidney disease). Systematic definition of these conditions was realized by amalgamating self-reported information furnished by participants and incorporating a diverse array of health-related records (Table S2). Detailed information pertaining to these records can be accessed on the UK Biobank website.

### Covariates

Based on the existing literature [7, 29, 30], we considered a set of covariates including age (year, in continuous), sex (male/female), ethnicity (white/others), educational levels (college/university, vocational, upper secondary, lower secondary, others, or unknown), Townsend deprivation index (in continuous, the lower quintile represents higher socioeconomic level), smoking status (never/current or previous), baseline cardiovascular diseases (yes/no), baseline hypertension (yes/no), medication use for cholesterol, blood pressure or diabetes (yes/no), and total energy intake (kcal/day, in continuous).

### Statistical analysis

We excluded participants with baseline T2D and/or DMC (*n* = 5,475), without 24-hour dietary assessments (*n* = 38,481), with only one 24-hour dietary assessment (*n* = 9,573), with more than two missing MEDLIFE items (*n* = 10), with extreme energy intake (< 800 or > 4200 kcal/day for male and < 600 or > 3500 kcal/day for female) (*n* = 78), with missing data for calculating VAT (*n* = 1,338). To mitigate the influence of outlier values in VAT, we opted to exclude individuals whose VAT mass fell below the 1st percentile or exceeded the 99th percentile (*n* = 208) [31]. Participants with missing data on covariates (*n* = 123) were also excluded. Finally, 11,267 participants were included in our study (Fig. 1).

Baseline characteristics among quartiles of the MEDLIFE score and VAT mass were subjected to comparisons utilizing either ANOVA or the chi-squared test. The evaluation of the association between MEDLIFE and VAT with the incidence of T2D, DMC, and specifically, diabetic nephropathy (considering the limited occurrence of other DMC) was carried out using Cox proportional hazards models. MEDLIFE index was modeled with a 2-point increment, while VAT was modeled per standard deviation (SD) increment. Both MEDLIFE Index and VAT mass were categorized into quartiles, with the first quartile (indicating the lowest adherence) serving as the reference. *P*-trends were computed using MEDLIFE quartiles and VAT quartiles as a continuous variable. Three progressively adjusted models were employed: Model 1, adjusted for age, sex, education, race, and deprivation index; Model 2, further adjusted for smoking status, baseline cardiovascular diseases, baseline hypertension, medication use for cholesterol, blood pressure or diabetes, and total energy intake; Model 3, further adjusting for MEDLIFE score or VAT. Combinations of MEDLIFE and VAT categories were used to collectively classify participants into 16 strata. Participants with the highest category of VAT and in the lowest category of MEDLIFE were used as the reference group for subsequent analyses. The investigation of nonlinear trends in the prevalence of T2D and associated diabetic complications employed the utilization of restricted cubic splines. Specifically, three knots were strategically positioned at centiles 10, 50, and 90 within the MEDLIFE index and VAT, with a MEDLIFE of 1 point and VAT mass of 1300 gram (the median) as references. The proportional hazards assumption was assessed for each analysis, and all analyses demonstrated conformity with the proportional hazard assumption.

To ascertain the robustness of our study, we conducted a series of sensitivity analyses to replicate and extend the main analysis. Firstly, accounting for competing mortality risk, we employed the Fine-Gray model to assess the associations. Secondly, to address potential reverse causality, we excluded cases diagnosed within the initial two years of follow-up for each outcome. We analyzed the association of MEDLIFE and outcomes by replicating assessments for each MEDLIFE block while adjusting for the remaining blocks. Additionally, we conducted analyses for individual MEDLIFE items, adjusting for the MEDLIFE index while excluding the corresponding item. For analysing the association of MEDLIFE with the incident outcomes, we restricted the analysis to individuals with a minimum of three diet assessments (*N* = 6,997) to explore potential deviations from usual dietary patterns. Furthermore, we systematically excluded each item from the MEDLIFE index, one at a time, to investigate whether a single item was driving the main association. To mitigate the potential mischaracterization of the study cohort, we augmented our analytical approach by replicating the primary analyses specifically for subjects meeting the criteria for prediabetes as delineated by HbA1c levels within the range of 5.7–6.4% (39–47 mmol/mol).

Hazard ratios (HRs) and 95% confidence intervals (CIs) were used to evaluate the associations, and *P* < 0.05 was considered as statistically significant. All statistical analyses were conducted with R (version 4.3.0).

## Results

Baseline characteristic of included individuals with prediabetes (*n* = 11,267; 48.98% male; mean age, 59.47 years) within categories of MEDLIFE and VAT were displayed in Table 1. Individuals with higher VAT quartile of vat were more likely to be male, lower educated, smoker (current or previous), higher deprived, more energy intake, higher prevalence of hypertension and cardiovascular diseases, and lower MEDLIFE index score. Participants with higher MELIFE quartile were more likely to be older, female, higher educated, lower deprived, non-smoker, less energy intake, less vat mass. Over a median follow-up duration of 13.77 years, a total of 1439 participants developed T2D and a cumulative total of 711 participants encountered at least one occurrence of a DMC (60 diabetic retinopathy cases, 87 diabetic neuropathy cases, and 585 diabetic kidney disease cases).

**Table 1:** Baseline characteristics

	Overall	MEDLIFE Index				VAT mass			
		Q1 (1–8)	Q2 (9)	Q3 (10–11)	Q4 (12–21)	Q1 (< 739)	Q2 (739–1296)	Q3 (1297–2001)	Q4 (> 2001)
**Number of participants, n**	11,267	4032	1775	3188	2272	2759	2874	2874	2760
**Age, mean (SD)**	59.47 (6.56)	58.97 (6.84)	59.38 (6.59)	59.67 (6.45)	60.17 (6.08)	58.84 (6.56)	59.95 (6.33)	59.63 (6.63)	59.44 (6.67)
**Male, n (%)**	5519 (49.0)	2221 (55.1)	908 (51.2)	1516 (47.6)	874 (38.5)	367 (13.3)	952 (33.1)	1810 (63.0)	2390 (86.6)
**White, n (%)**	10,264 (91.1)	3723 (92.3)	1625 (91.5)	2876 (90.2)	2040 (89.8)	2485 (90.1)	2589 (90.1)	2619 (91.1)	2571 (93.2)
**Education, n (%)**									
College/university	4655 (41.3)	1413 (35.0)	730 (41.1)	1393 (43.7)	1119 (49.3)	1324 (48.0)	1182 (41.1)	1189 (41.4)	960 (34.8)
Vocational	3826 (34.0)	1474 (36.6)	602 (33.9)	1022 (32.1)	728 (32.0)	927 (33.6)	1025 (35.7)	947 (33.0)	927 (33.6)
Upper secondary	1026 (9.1)	461 (11.4)	157 (8.8)	267 (8.4)	141 (6.2)	159 (5.8)	201 (7.0)	264 (9.2)	402 (14.6)
Lower secondary	654 (5.8)	204 (5.1)	103 (5.8)	207 (6.5)	140 (6.2)	141 (5.1)	179 (6.2)	165 (5.7)	169 (6.1)
Others	1061 (9.4)	460 (11.4)	173 (9.7)	291 (9.1)	137 (6.0)	198 (7.2)	272 (9.5)	298 (10.4)	293 (10.6)
Unknown	45 (0.4)	20 (0.5)	10 (0.6)	8 (0.3)	7 (0.3)	10 (0.4)	15 (0.5)	11 (0.4)	9 (0.3)
**TDI, mean (SD)**	− 1.57 (2.88)	− 1.45 (2.96)	− 1.63 (2.86)	− 1.63 (2.84)	− 1.67 (2.80)	− 1.66 (2.80)	− 1.61 (2.83)	− 1.54 (2.97)	− 1.48 (2.93)
**Smoking status, n (%)**									
Never	5782 (51.3)	1970 (48.9)	882 (49.7)	1675 (52.5)	1255 (55.2)	1698 (61.5)	1616 (56.2)	1388 (48.3)	1080 (39.1)
Previous	4353 (38.6)	1558 (38.6)	682 (38.4)	1243 (39.0)	870 (38.3)	763 (27.7)	992 (34.5)	1193 (41.5)	1405 (50.9)
Current	1132 (10.0)	504 (12.5)	211 (11.9)	270 (8.5)	147 (6.5)	298 (10.8)	266 (9.3)	293 (10.2)	275 (10.0)
**Total energy intake, kal/day, mean (SD)**	2080.68 (503.05)	2161.58 (499.25)	2071.22 (505.29)	2034.58 (495.13)	2009.19 (499.72)	1982.16 (448.55)	2020.96 (490.47)	2116.52 (504.21)	2204.02 (535.74)
**Baseline hypertension, n (%)**	1046 (9.3)	389 (9.6)	169 (9.5)	301 (9.4)	187 (8.2)	104 (3.8)	201 (7.0)	322 (11.2)	419 (15.2)
**Baseline CVDs, n (%)**	841 (7.5)	298 (7.4)	138 (7.8)	254 (8.0)	151 (6.6)	77 (2.8)	153 (5.3)	266 (9.3)	345 (12.5)
**Medication use, n (%)**	4194 (37.2)	1529 (37.9)	705 (39.7)	1166 (36.6)	794 (34.9)	522 (18.9)	951 (33.1)	1260 (43.8)	1461 (52.9)
**MEDLIFE Index, mean (SD)**	9.49 (2.51)	6.90 (1.18)	9.00 (0.00)	10.44 (0.50)	13.14 (1.36)	10.16 (2.51)	9.62 (2.47)	9.29 (2.50)	8.91 (2.40)
**Block 1 of MEDLIFE, mean (SD)**	3.40 (1.61)	2.21 (1.09)	3.06 (1.08)	3.75 (1.11)	5.28 (1.38)	3.70 (1.68)	3.42 (1.62)	3.30 (1.60)	3.18 (1.51)
**Block 2 of MEDLIFE, mean (SD)**	3.21 (1.17)	2.44 (1.00)	3.10 (0.94)	3.52 (0.94)	4.21 (0.95)	3.39 (1.14)	3.28 (1.15)	3.15 (1.18)	3.00 (1.19)
**Block 3 of MEDLIFE, mean (SD)**	2.89 (1.14)	2.26 (1.03)	2.84 (1.00)	3.17 (0.98)	3.65 (1.04)	3.07 (1.13)	2.92 (1.14)	2.83 (1.14)	2.73 (1.14)
**MEDLIFE Index (category)**									
Q1 (1–8)	4032 (35.8)	–	–	–	–	706 (25.6)	964 (33.5)	1130 (39.3)	1232 (44.6)
Q2 (9)	1775 (15.8)	–	–	–	–	407 (14.8)	462 (16.1)	448 (15.6)	458 (16.6)
Q3 (10–11)	3188 (28.3)	–	–	–	–	865 (31.4)	838 (29.2)	790 (27.5)	695 (25.2)
Q4 (12–21)	2272 (20.2)	–	–	–	–	781 (28.3)	610 (21.2)	506 (17.6)	375 (13.6)
**VAT mass, g, mean (SD)**	1429.79 (859.51)	1593.65 (865.87)	1467.48 (872.40)	1364.29 (836.10)	1201.48 (807.19)	453.13 (185.70)	1009.31 (159.07)	1622.61 (200.19)	2643.17 (514.26)
**VAT mass (category)**									
Q1 (< 739)	2759 (24.5)	706 (17.5)	407 (22.9)	865 (27.1)	781 (34.4)	–	–	–	–
Q2 (739–1296)	2874 (25.5)	964 (23.9)	462 (26.0)	838 (26.3)	610 (26.8)	–	–	–	–
Q3 (1297–2001)	2874 (25.5)	1130 (28.0)	448 (25.2)	790 (24.8)	506 (22.3)	–	–	–	–
Q4 (> 2001)	2760 (24.5)	1232 (30.6)	458 (25.8)	695 (21.8)	375 (16.5)	–	–	–	–

Q1, Quartile 1; Q2, Quartile 2; Q3, Quartile 3; Q4, Quartile 4; MEDLIFE, Mediterranean lifestyles. VAT: visceral adipose tissue; MEDLIFE: Mediterranean lifestyle; TDI, Townsend deprivation index; CVDs, cardiovascular diseases

Block 1: Mediterranean food consumption; Block 2: Mediterranean dietary habits; Block 3: Physical activity, rest, social habits, and conviviality

**Fig. 2:**
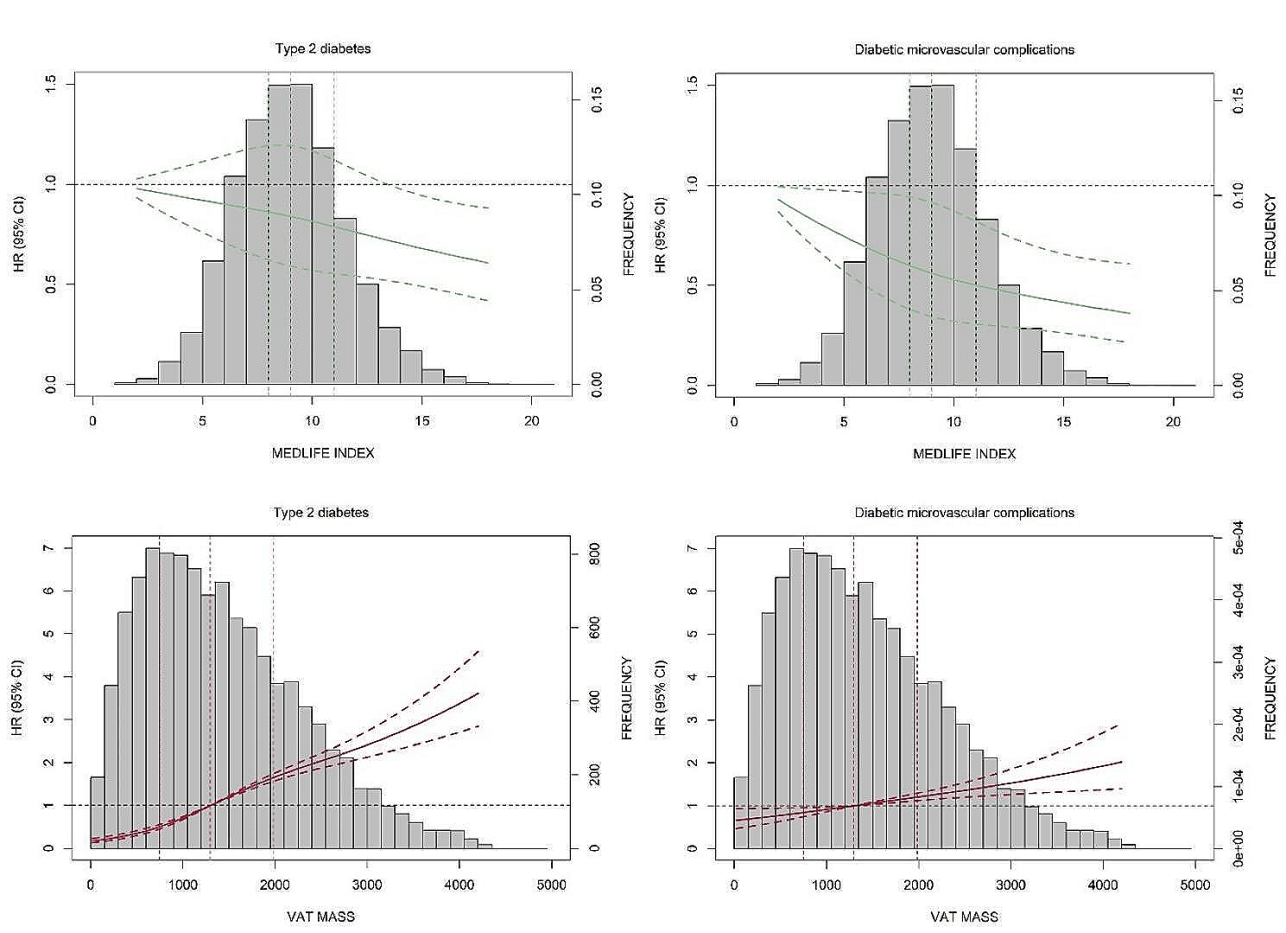
Association between MEDLIFE INDEX and VAT with T2D and any microvascular complications. Plotted values are HR (95% CI) from a restricted cubic spline Cox regression model, with a MEDLIFE INDEX of 1 point and VAT mass of 1300 gram (the median) as references. The vertical dashed line represents the quartile line. Adjusted for age, sex, education, race, and deprivation index, smoking status, baseline cardiovascular diseases, baseline hypertension, medication use for cholesterol, blood pressure or diabetes, and total energy intake, and MEDLIFE score or VAT. HR, Hazard Ratios; CI, Confidence Intervals; T2D, Type 2 diabetes; VAT: visceral adipose tissue; MEDLIFE: Mediterranean lifestyle

In the most adjusted model, per increment (2 points) in the MEDLIFE score decreased the risk of T2D (HR, 95% CI: 0.94, 0.90–0.99) and any DMC (0.89, 0.83–0.94). Compared to the lowest quartile, the highest quartile demonstrates a risk reduction of 16% and 31%, for incident T2D (0.84, 0.71–0.98) and incident any DMC (0.69, 0.56–0.86), respectively (Table 2). Per SD VAT increment increased presented a risk increase of 76% and 28% for T2D (1.76, 1.66–1.87) and any DMC (1.28, 1.16–1.40), respectively. Compared to the lowest quartile, the highest quartile increased the risk of T2D (5.95, 4.72–7.49) and incident any DMC (1.79, 1.36–2.35) (Table 3). An inverse dose-response relationship was evident between MEDLIFE and T2D and microvascular complications, whereas a dose-response relationship was observed between VAT and all outcomes (*P* for trend < 0.05) (Tables 2 and 3). For specific microvascular complications, higher MEDLIFE decreased the risk of diabetic nephropathy (0.85, 0.79–0.91) and higher VAT elevated the risk of diabetic nephropathy (1.25, 1.13–1.39) (Table S3). Additionally, in the context of restricted cubic spline analysis, a nearly linear dose-response pattern was discerned across all associations (Fig. 2 and Fig. S1).

**Table 2:** The association of MEDLIFE index with risk of T2D and microvascular complications in individuals with prediabetes

		Q1	Q2	Q3	Q4	*P* for trend	Per two points increase
			HR (95% CI)	HR (95% CI)	HR (95% CI)		HR (95% CI)
T2D	Model 1	Ref	1.01 (0.87, 1.17)	0.87 (0.76, 0.99)*	0.75 (0.64, 0.87)*	< 0.001	0.91 (0.87, 0.95)*
	Model 2	Ref	0.99 (0.85, 1.15)	0.86 (0.76, 0.98)*	0.75 (0.64, 0.88)*	< 0.001	0.91 (0.87, 0.95)*
	Model 3	Ref	1.01 (0.87, 1.18)	0.94 (0.82, 1.07)	0.84 (0.71, 0.98)*	0.031	0.94 (0.90, 0.99)*
Any microvascular complication	Model 1	Ref	0.85 (0.68, 1.05)	0.74 (0.61, 0.89)*	0.66 (0.53, 0.82)*	< 0.001	0.87 (0.82, 0.93)*
	Model 2	Ref	0.83 (0.67, 1.03)	0.73 (0.61, 0.88)*	0.66 (0.53, 0.82)*	< 0.001	0.87 (0.82, 0.93)*
	Model 3	Ref	0.84 (0.68, 1.05)	0.76 (0.63, 0.91)*	0.69 (0.56, 0.86)*	< 0.001	0.89 (0.83, 0.94)*

Model 1: Adjusted for age, sex, education, race, and deprivation index; Model 2: Further adjusted for smoking status, baseline cardiovascular diseases, baseline hypertension, medication use for cholesterol, blood pressure or diabetes, and total energy intake, Model 3: Further adjusted for VAT

*P < 0.05; Q1, Quartile 1; Q2, Quartile 2; Q3, Quartile 3; Q4, Quartile 4; ref, reference; HR, Hazard Ratios; CI, Confidence Intervals; T2D, Type 2 diabetes; VAT: visceral adipose tissue; MEDLIFE: Mediterranean lifestyle

**Table 3:** The association of VAT with risk of T2D and microvascular complications in individuals with prediabetes

		Q1	Q2	Q3	Q4	*P* for trend	Per SD increase
			HR (95% CI)	HR (95% CI)	HR (95% CI)		HR (95% CI)
T2D	Model 1	Ref	2.36 (1.88, 2.96)*	4.17 (3.35, 5.20)*	7.19 (5.74, 9.01)*	< 0.001	1.88 (1.77, 1.99)*
	Model 2	Ref	2.23 (1.78, 2.80)*	3.72 (2.98, 4.64)*	6.12 (4.87, 7.70)*	< 0.001	1.78 (1.67, 1.89)*
	Model 3	Ref	2.20 (1.75, 2.76)*	3.64 (2.91, 4.55)*	5.95 (4.72, 7.49)*	< 0.001	1.76 (1.66, 1.87)*
Any microvascular complication	Model 1	Ref	1.15 (0.90, 1.48)	1.59 (1.23, 2.04)*	2.38 (1.83, 3.10)*	< 0.001	1.42 (1.30, 1.56)*
	Model 2	Ref	1.07 (0.83, 1.37)	1.36 (1.05, 1.75)*	1.91 (1.45, 2.50)*	< 0.001	1.30 (1.19, 1.43)*
	Model 3	Ref	1.04 (0.80, 1.33)	1.29 (0.99, 1.66)	1.79 (1.36, 2.35)*	< 0.001	1.28 (1.16, 1.40)*

Model 1: Adjusted for age, sex, education, race, and deprivation index; Model 2: Further adjusted for smoking status, baseline cardiovascular diseases, baseline hypertension, medication use for cholesterol, blood pressure or diabetes, and total energy intake, Model 3: Further adjusted for MEDLIFE Index

*P < 0.05; Q1, Quartile 1; Q2, Quartile 2; Q3, Quartile 3; Q4, Quartile 4; ref, reference; HR, Hazard Ratios; CI, Confidence Intervals; T2D, Type 2 diabetes; VAT: visceral adipose tissue; MEDLIFE: Mediterranean lifestyle

**Fig. 3:**
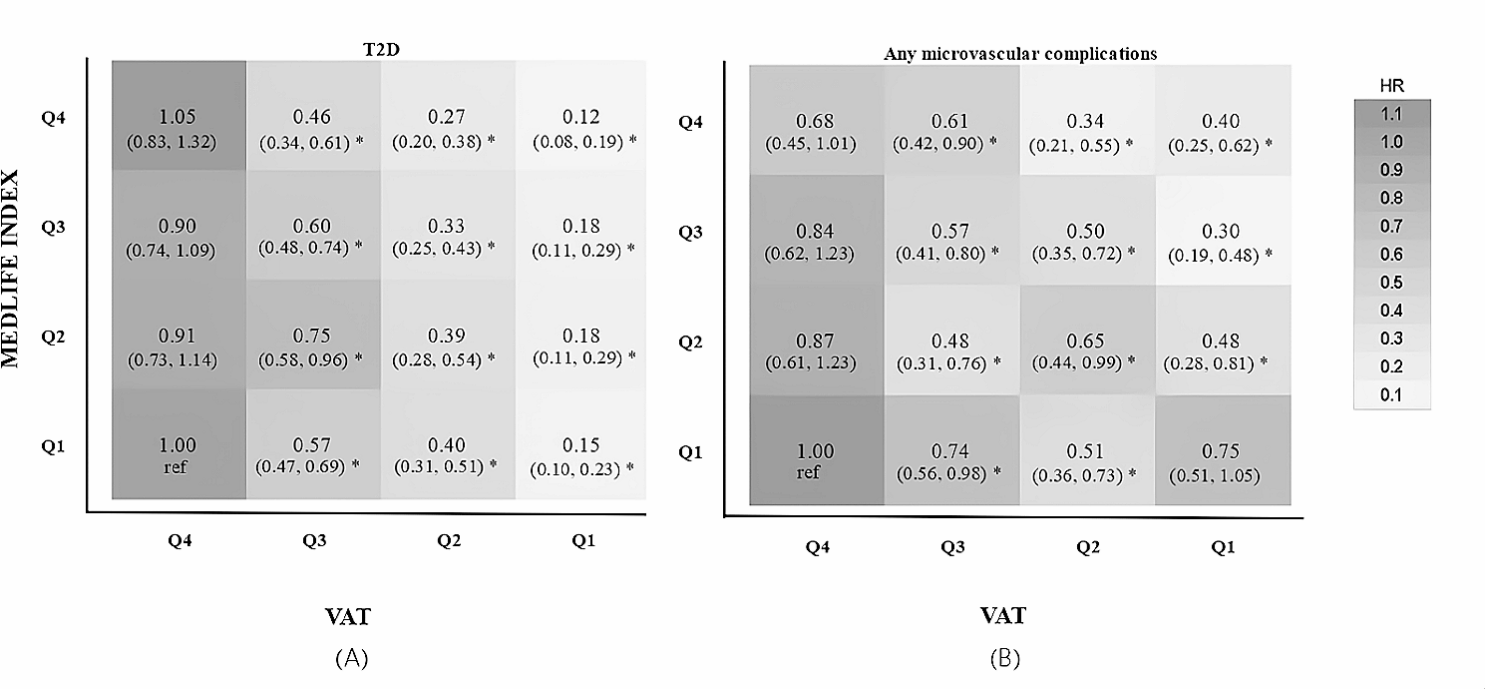
Associations of combinations of MEDLIFEN Index and VAT mass with incident T3D and microvascular complications. Estimated by multivariable-adjusted HRs by use of Cox regression analysis with the highest category of VAT and in the lowest category of MEDLIFE as the reference. Adjusted for age, sex, education, race, and deprivation index, smoking status, baseline cardiovascular diseases, baseline hypertension, medication use for cholesterol, blood pressure or diabetes, and total energy intake. Ref, reference.; HR, Hazard Ratios; CI, Confidence Intervals; T3D, Type 3 diabetes; VAT: visceral adipose tissue; MEDLIFE: Mediterranean lifestyle

Figure 3A illustrates associations between cross-classified MEDLIFE and VAT categories with T2D and microvascular complications. Using the lowest MEDLIFE quartile and highest VAT quartile as a reference, the lowest T2D risk was found in individuals with lowest VAT and highest MEDLIFE (HR 0.12; 95% CI 0.08–0.19). The high MEDLIFE index was associated with reduced T2D risk regardless of VAT category, except in individuals with the highest quartile of VAT, where this association was not observed. Additionally, lower VAT levels mitigated the risk associated with a low MEDLIFE index. Similar trends were observed in microvascular complications, except for the combination of lowest MEDLIFE and lowest VAT, which didn’t significantly reduce microvascular complication risk (HR 0.75, 95% CI 0.51–1.05) (Fig. 3B). Results were consistent in the analysis of diabetic nephropathy (Figure S2).

After excluding each item from the MEDLIFE index, the associations of MEDLIFE with T2D and microvascular risk remained unchanged (Table S4). The association between each block (physical activity, rest, social habits, and conviviality) and the respective outcomes was examined. Incremental increases of two points in blocks two and three were associated with reduced T2D risk, with HRs of 0.87 (0.79–0.95) and 0.91 (0.83–0.99), respectively. Additionally, two-point increments in block one and three correlated with decreased microvascular complications risk, showing HRs of 0.89 (0.80–0.98) and 0.87 (0.77–0.99) (Table S5). The results of our sensitivity analyses demonstrated the robustness of the majority of findings, taking into account the competing risk of death, exclusion of events within the first two years of follow-up, exclusion of participants with baseline cardiovascular disease, inclusion exclusively of participants with a minimum of three 24-hour dietary assessments, and only included subjects meeting the criteria for prediabetes as delineated by HbA1c levels (Table S6 to Table S13).

## Discussion

In this large cohort analysis of individuals with prediabetes, high adherence to a MEDLIFE correlated with decreased risks of T2D and microvascular complications, while elevated VAT mass increased incident risk. Overall, except for the highest VAT category, adherence to a MEDLIFE was associated with reduced incidences of T2D and microvascular complications linked to high VAT. Lower VAT levels appeared to counteract the increased risk of T2D associated with low adherence to a MEDLIFE. These findings suggest that both adherence to a healthy lifestyle, such as a MEDLIFE, and reduced VAT mass are essential for preventing secondary complications in individuals with prediabetes.

Lifestyle modifications constituted the foundational approach for managing prediabetes [32]. Several prior lifestyle intervention studies exhibited inconsistency in results due to biases and limitations in sample sizes. Post-trial analysis of the Daqing Diabetes Prevention Outcomes Study in individuals with prediabetes revealed that lifestyle intervention reduced microvascular, cardiovascular, and all-cause mortality [33]. However, findings from the Diabetes Prevention Program Outcomes Study and Look AHEAD (Action for Health in Diabetes) did not show a protective effect of lifestyle intervention on these outcomes [34]. In a recent study with over 2 million participants, lifestyle intervention, including personalized support, healthier diets, and increased physical activity, led to an average 15 mg/dL reduction in blood glucose levels among individuals with prediabetes [5].

Studies above predominantly concentrate on alterations in diet and physical activity, lacking a more intricate evaluation of lifestyle. The MEDLIFE Index integrates diverse diets and lifestyle behaviors, potentially generating synergistic effects. The Mediterranean dietary pattern, rich in vitamins, minerals, and polyphenols, exhibits antioxidant and anti-inflammatory properties, improving HbA1c levels, fasting glucose, and insulin resistance index [35]. High intake of fiber, adequate consumption of tea and coffee, and polyunsaturated fatty acids from nuts and fish reduces insulin resistance, postprandial blood glucose fluctuations, inflammation, and weight gain [36–38]. However, in the analysis of the MEDLIFE block one, we did not observe a statistically significant reduction in T2D risk. This lack of significance may be attributed to the omission of components characteristic of the Mediterranean diet, particularly olive oil, in our MEDLIFE index for this analysis. Olive oil, with its unique lipid composition abundant in monounsaturated fatty acids, notably oleic acid, along with various bioactive compounds, exhibits protective effects on lipid metabolism, inflammation, endothelial function, and oxidative stress [39, 40]. Previous epidemiological research has associated olive oil consumption with a 22% decrease in the relative risk of T2D [41]. Additionally, the consumption of Mediterranean Diets supplemented with virgin olive oil reduced diabetes rates by 51% (0.49 [0.25–0.97]) [42]. The exclusion of olive oil from our analysis may potentially underestimate the impact of a MEDLIFE. However, our main findings remain consistent even when other items were excluded from the MEDLIFE score. The benefits of a MEDLIFE can be attained without specific features of the Mediterranean diet, emphasizing a holistic lifestyle pattern over reliance on individual components. Physical activity reduces systemic inflammatory biomarkers, reactive oxygen species, enhances antioxidant proteins, mitigates mitochondrial fission, and improves insulin sensitivity [43]. Social and entertainment factors in MEDLIFE correlate with reduced T2D risk [44] and lower T2D mortality [45]. Overall, MEDLIFE components may synergistically affect systemic inflammation, oxidative stress, glycemic control, satiety, insulin resistance, and weight maintenance, potentially preventing T2D and microvascular complications.

Obesity plays a significant role in diabetes onset [16], with studies indicating that the heightened metabolic and cardiovascular risks associated with obesity are closely tied to body fat distribution rather than total body fat mass [46]. Commonly used obesity indicators, such as BMI and waist circumference (WC), lack precision in accounting for local fat deposition heterogeneity and distinguishing between lean body mass and fat body mass [17]. Notably, abdominal visceral fat accumulation, a crucial phenotype related to complications, is inadequately indicated by both BMI and WC [47, 48]. Previous investigations have consistently delineated associations between the accrual of abdominal fat, notably VAT in contrast to SAT, and the susceptibility to diverse DMC, with visceral fat obesity emerging as an independent predictor of diabetes [17, 49, 50]. Additionally, studies demonstrated between elevated visceral fat area, or composite indicators reflecting visceral fat function (i.e., VAI and LAP), and the occurrence of T2D and related microvascular complications [21, 51]. The accumulation of visceral fat is associated with conditions such as insulin resistance, T2D, and hypertension, significantly heightening the risk of DMC [52]. In addition, dysfunction in adipose tissue, stemming from excessive visceral adiposity and ectopic fat accumulation, serves as a primary driver of insulin resistance syndrome [53, 54]. Despite this, there is a paucity of studies investigating the connection between VAT mass and these endpoints. In our findings, VAT mass demonstrated a linear association with the risk of both T2D and DMC. The causal relationships between VAT mass and the development of T2D and other cardiometabolic diseases have also been substantiated by a recent Mendelian randomization study [28].

Clinical randomized trials have previously illustrated that the Mediterranean diet can alleviate cardiovascular risk linked to abdominal obesity, as evidenced by abdominal obesity indices such as WC and waist-to-hip ratio [55], providing further support for our research. Additionally, other studies have suggested that reducing abdominal fat through diet is linked to decreased systemic free fatty acid flux and improved insulin sensitivity [56]. In our study, a high MEDLIFE index reduced the risk of T2D and related DMC associated with visceral fat accumulation, except in the highest quartile. This may be attributed to the MEDLIFE index’s potential to diminish the inflammatory response [35]. However, despite this reduction, excessive accumulation of visceral fat can still lead to irreversible metabolic disorders within visceral white adipose tissue, characterized by abnormal mitochondrial morphology and function [57]. The implications of disrupted mitochondrial dynamics are closely associated with mitochondrial function and the onset and progression of diabetic complications [58]. Moreover, surplus visceral adipose tissue can inflict irreversible structural and functional harm on macrophages, preadipocytes, mature adipocytes, and endothelial cells, resulting in heightened metabolic activity, concurrent production of oxygen free radicals, mitochondrial dysfunction, and susceptibility to DNA damage [59, 60]. Additionally, it may hinder the physiological remodeling of the extracellular matrix in visceral adipose tissue, leading to decreased tissue plasticity and ultimately exacerbating local inflammatory responses [61]. These disorders, including mitochondrial dynamics abnormalities, are intricately linked to the emergence and progression of inflammatory reactions and diabetic complications [58].

Our study has several strengths, to the best of our knowledge, this is the first extensive cohort study involving individuals with prediabetes, specifically scrutinizing the individual and combined associations of the MEDLIFE index and VAT mass with subsequent T2D and related microvascular disease. The distinctive aspects of our study encompass the incorporation of a comprehensive Mediterranean lifestyle, surpassing sole emphasis on the Mediterranean dietary pattern. Additionally, the utilization of visceral fat mass as an adiposity metric, distinct from BMI, contributes to the methodological innovation. Furthermore, our research introduces the examination of combined strata of MEDLIFE and VAT mass, providing a nuanced perspective on their joint impact. We employed a minimum of two dietary assessments in calculating the MEDLIFE index, and subsequent analyses using three or more assessments yielded consistent results, indicating the reliability of our findings. However, our study has limitations, including the inability to access data on certain MEDLIFE components due to UK Biobank data constraints, potentially leading to underestimation of associations. Additionally, voluntary participant recruitment may introduce bias, with previous analyses indicating that participants completing more dietary assessments tend to be older and more educated than the general UK Biobank population [62]. Furthermore, while the VAT prediction model utilized may not offer the same accuracy as imaging results, its feasibility has been demonstrated in previous large-scale population studies [31].

In conclusion, high adherence to MEDLIFE decreased the risk of T2D and MDC in individuals with prediabetes, whereas high VAT mass elevated the risk of these conditions in individuals with prediabetes. However, the risk associated with high VAT mass can be partially mitigated by adhering to a high MEDLIFE. The potential adaptability of the Mediterranean lifestyle to various populations presents a promising approach for fostering healthy behaviors and thereby preventing adverse health outcomes, such as T2D.

### Electronic supplementary material

Below is the link to the electronic supplementary material.

Supplementary Material 1

## Data Availability

All data utilized in this study are accessible through the official repository of the UK Biobank, which can be found at https://www.ukbiobank.ac.uk/.
