# Supplementary material for: Combined associations of visceral adipose tissue and adherence to a Mediterranean lifestyle with T2D and diabetic microvascular complications among individuals with prediabetes
Source: Cardiovasc Diabetol. 2024 Jun 12;23:201. doi: 10.1186/s12933-024-02284-1 (PMC11170917; doi:10.1186/s12933-024-02284-1)
Supplement: Supplementary file 1 — Supplementary Material 1 [file 12933_2024_2284_MOESM1_ESM.docx]

**Tables and figures lengths**

**Table S1.** An adapted version of the Mediterranean Lifestyle (MEDLIFE) Index tailored for implementation in the UK Biobank Study (n=11,267).

**Table S2.** UKB filed code of definition of baseline diabetes and related complications analysed in this study.

**Table S3.** The association of MEDLIFE index and VAT with risk of diabetic nephropathy in participants with prediabetes.

**Table S4.** The association between each MEDLIFE Item and the risk of T2D and microvascular complications in participants with prediabetes, excluding items from the score.

**Table S5.** The association between MEDLIFE blocks and the risk of T2D and microvascular complications in participants with prediabetes.

**Table S6.** The association of MEDLIFE index with risk of T2D and microvascular complications in participants with prediabetes, considering death as competing event.

**Table S7.** The association of VAT with risk of T2D and microvascular complications in participants with prediabetes, considering death as competing event.

**Table S8.** The association of MEDLIFE index with risk of T2D and microvascular complications in participants with prediabetes, excluding cases within first two years of follow-up.

**Table S9.** The association of VAT with risk of T2D and microvascular complications in participants with prediabetes, excluding cases within first two years of follow-up.

**Table S10.** The association of MEDLIFE index with risk of T2D and microvascular complications in participants with prediabetes, among participants who completed three or more 24 hours dietary assessments.

**Table S11.** The association of VAT with risk of T2D and microvascular complications in participants with prediabetes, among participants who completed three or more 24 hours dietary assessments.

**Table S12.** The association of MEDLIFE index with risk of T2D and microvascular complications, among subjects meeting the criteria for prediabetes as delineated by HbA1c levels.

**Table S13.** The association of VAT with risk of T2D and microvascular complications, among subjects meeting the criteria for prediabetes as delineated by HbA1c levels.

**Fig S1.** Association between MEDLIFE INDEX and VAT with diabetic nephropathy.

**Fig. S2** Associations of combinations of MEDLIFEN Index and VAT mass with incident diabetic nephropathy.

**Table S1.** An adapted version of the Mediterranean Lifestyle (MEDLIFE) Index tailored for implementation in the UK Biobank Study (n=11,312).

| **Index items** | **Components (serving size)** | **Criteria for 1 point** | **% scoring 1 point *** |
| --- | --- | --- | --- |
| Block 1: *Mediterranean food consumption* | | |  |
| Sweets | Cookies, chocolate cookies, pastries, donuts, homemade baked goods, store-bought baked goods, muffins (50 g), chocolates (30 g). | ≤ 2 serv/wk | 15.68 |
| Red meat | Beef, pork, lamb (125 g). | ≤ 2 serv/wk | 49.62 |
| Processed meat | Sausage, soft spicy sausage, bacon, cured ham, cooked ham, hamburger, liver, organ meats, pâté (42.5 g). | ≤ 1 serv/wk | 44.86 |
| Eggs | Eggs (1 unit = 65 g). | ≥ 2 to ≤ 4 serv/wk | 15.31 |
| Legumes | Lentils, beans, chickpeas, peas (150 g cooked). | ≥ 2 serv/wk | 18.81 |
| White meat | Chicken/turkey with skin, chicken/turkey without skin, rabbit (125 g). | ≤ 2 serv/wk | 64.38 |
| Fish/seafood | White fish, fatty fish, codfish, salted or smoked fish, shrimp, octopus, calamari, oysters and shellfish (125 g). | ≥ 2 serv/wk | 38.44 |
| Potatoes | Baked or boiled potatoes (150 g). | ≤ 3 serv/wk | 34.91 |
| Low-fat dairy products | Skim milk, low-fat milk (200 ml), low fat yogurt (125 g), fresh soft cheese (50 g). | 1.5-2.5 serv/d | 7.87 |
| Nuts | Almonds, peanuts, hazelnuts, walnuts (30 g). | ≥ 3 serv/wk | 18.71 |
| Fruit | Orange, banana, apple, pear, kiwi, mango, avocado, peach, apricot, nectarine, clementine, strawberry, cherries, plums, figs, grapes, watermelon, melon (160 g), dates and dried fruits (30 g). | ≥ 3 serv/d | 9.59 |
| Vegetables | Spinach, cauliflower, broccoli, lettuce, carrot, squash, green beans, eggplant, zucchini, cucumber, pepper, asparagus, gazpacho, garden salad, tomato, other vegetables (236.59 g for green leafy, half for others) *(excluding potatoes).* | ≥ 2 serv/d | 21.67 |
| Block 2: *Mediterranean* d*ietary habits* | | | |
| Wine | Red/white wine (1 glass, 100 ml). | Women: > 0 to ≤ 1 serv/d  Men: > 0 to ≤ 2 serv/d | 25.17 |
| Limit salt at meals | Do you add salt to your food? (*After cooking*). | “Never” or “Sometimes” | 84.03 |
| Low salt consumption | Low sodium-to-potassium ratio and low sodium consumption. | Na/K ratio ≤ 0.57  Na ≤ 2.000 mg/d | 46.03 |
| Preference for whole grain products | Fiber from whole grain cereals. | > 6 g/d fiber from cereals | 10.59 |
| Snacks | Potato chips, popcorn, or other chips (50 g). | ≤ 1 serv/wk | 58.26 |
| Healthy beverages consumption | Coffee, decaffeinated coffee (1 cup = 50 ml) or tea (1 cup = 250 ml). | ≥ 1 to ≤ 4 serv/d | 71.08 |
| Limited consumption of sugar-sweetened beverages | Sugar-sweetened beverages + juice (150 ml). | < 1/wk | 24.43 |
| Block 3: *Physical activity, rest social habits and conviviality* | | | |
| Physical activity | Brisk walking, jogging, running, climbing stairs, bicycling, stationary cycling, swimming, dance, aerobic exercise, martial arts, gymnastics, gardening, tennis, soccer, skiing, ice skating, team sports, and other physical activities or sports. | ≥ 150 min of moderate, 75 min vigorous, or an equivalent combination | 75.00 |
| Nap | Napping throughout the week. | “Usually” or “Sometimes” if night-time sleep is adequate | 39.38 |
| Hours of sleep | Sleeping throughout the week. | 6-8 h/d | 88.82 |
| Limit sedentary activities | Watching TV, using internet or driving. | ≤ 2 h/d | 18.65 |
| Collective sports | Playing soccer, tennis, squash, basketball, or other team sports, running in group, jogging; etc. | ≥ 1 h/wk | 29.12 |
| Socializing with friends or family | Frequency on what you see family (different from whom you live with) or friends (home, church, bars…). | ≥ 3 times/wk | 40.67 |
| Four items (Sofrito, Olive oil, limiting snacking between meals, eating in company) were omitted from the analysis due to the absence of pertinent data in the records of the UK Biobank.  Abbreviations: N, total number of participants; serv, serving; d, day, wk, week; Tbsp, tablespoons; TV, television.  * The proportion of the study population that acquires one point for each respective item. | | | |

**Table S2.** UKB filed code of definition of baseline diabetes and related complications analysed in this study.

| **Disease** | **ICD10** | **ICD9** | **Self-reported non-cancer illness** |
| --- | --- | --- | --- |
| **Type 2 diabetes** | E110/E119/E121/E129/E130/E139/E140/E149 | 25000/25001/25009/25010/25019/25029/2503/2504/2505/25099 | 20002(1220/1222/1223) |
| **Diabetic retinopathy** | E113/E123/E133/E143/H280/H360 | 2504/3620 | 20002 (1607) |
| **Diabetic neuropathy** | E104/E114/E124/E134/E144/G590/G632/G990 | 2505/3572 | 20002 (1468) |
| **Diabetic kidney disease** | E102/E112/E122 E132/E142/N083 | 2053 | 20002 (1276) |

**Table S3.** The association of MEDLIFE index and VAT with risk of diabetic nephropathy in participants with prediabetes.

|  |  | **Q1** | **Q2** | **Q3** | **Q4** | ***P* for trend** | **Per one unit increase #** |
| --- | --- | --- | --- | --- | --- | --- | --- |
|  |  |  | **HR (95% CI)** | **HR (95% CI)** | **HR (95% CI)** |  | **HR (95% CI)** |
| **MEDLIFE** | **model 1** | ref | 0.78 (0.61, 0.99) * | 0.71 (0.58, 0.87) * | 0.56 (0.43, 0.72) * | <0.001 | 0.84 (0.78, 0.89) * |
|  | **model 2** | ref | 0.77 (0.61, 0.98) * | 0.71 (0.58, 0.87) * | 0.56 (0.44, 0.72) * | <0.001 | 0.84 (0.78, 0.89) * |
|  | **model 3** | ref | 0.78 (0.61, 0.99) * | 0.73 (0.60, 0.90) * | 0.59 (0.46, 0.75) * | <0.001 | 0.85 (0.79, 0.91) * |
|  |  |  |  |  |  |  |  |
| **VAT** | **model 1** | ref | 1.17 (0.89, 1.54) | 1.59 (1.20, 2.11) * | 2.46 (1.84, 3.29) * | <0.001 | 1.43 (1.29, 1.58) * |
|  | **model 2** | ref | 1.06 (0.80, 1.40) | 1.30 (0.98, 1.73) | 1.86 (1.38, 2.51) * | <0.001 | 1.28 (1.16, 1.43) * |
|  | **model 3** | ref | 1.01 (0.77, 1.34) | 1.21 (0.91, 1.61) | 1.70 (1.26, 2.30) * | <0.001 | 1.25 (1.13, 1.39) * |
| Adjusted for age, sex, education, race, and deprivation index, body mass index, smoking status, and total energy intake, duration of diabetes, diabetic medication use.  *, *P* < 0.05; Q1, Quartile 1; Q2, Quartile 2; Q3, Quartile 3; Q4, Quartile 4; ref, reference.; HR, Hazard Ratios; CI, Confidence Intervals; T2D, Type 2 diabetes.  # Per one unit for MEDLIFE equals two points of MEDLIFE score, while per one unit for VAT equals per SD of VAT. | | | | | | | |

**Table S4.** The association between each MEDLIFE Item and the risk of T2D and microvascular complications in participants with prediabetes, excluding items from the score.

|  | **T2D** | **Any microvascular complication** | **Diabetic nephropathy** |
| --- | --- | --- | --- |
|  | **HR (95% CI)** | **HR (95% CI)** | **HR (95% CI)** |
| **Sweets** | 0.90 (0.86, 0.94) * | 0.88 (0.82, 0.94) * | 0.84 (0.78, 0.90) * |
| **Red meat** | 0.90 (0.86, 0.94) * | 0.87 (0.81, 0.93) * | 0.84 (0.78, 0.90) * |
| **Processed meat** | 0.90 (0.86, 0.95) * | 0.87 (0.81, 0.93) * | 0.83 (0.77, 0.89) * |
| **Eggs** | 0.90 (0.86, 0.94) * | 0.88 (0.82, 0.93) * | 0.84 (0.79, 0.90) * |
| **Legumes** | 0.89 (0.85, 0.93) * | 0.87 (0.82, 0.93) * | 0.83 (0.78, 0.89) * |
| **White meat** | 0.90 (0.86, 0.94) * | 0.87 (0.82, 0.92) * | 0.83 (0.78, 0.89) * |
| **Fish/seafood** | 0.90 (0.86, 0.94) * | 0.88 (0.83, 0.94) * | 0.85 (0.79, 0.91) * |
| **Potatoes** | 0.90 (0.86, 0.95) * | 0.88 (0.82, 0.93) * | 0.84 (0.79, 0.90) * |
| **Low-fat dairy products** | 0.90 (0.86, 0.94) * | 0.88 (0.83, 0.94) * | 0.84 (0.79, 0.91) * |
| **Nuts** | 0.90 (0.86, 0.94) * | 0.86 (0.81, 0.92) * | 0.83 (0.77, 0.89) * |
| **Fruit** | 0.90 (0.86, 0.94) * | 0.87 (0.82, 0.93) * | 0.84 (0.78, 0.90) * |
| **Vegetables** | 0.90 (0.86, 0.94) * | 0.87 (0.82, 0.93) * | 0.83 (0.77, 0.89) * |
| **Wine** | 0.91 (0.87, 0.95) * | 0.87 (0.81, 0.92) * | 0.83 (0.78, 0.90) * |
| **Limit salt at meals** | 0.91 (0.87, 0.95) * | 0.88 (0.82, 0.93) * | 0.84 (0.78, 0.90) * |
| **Low salt consumption** | 0.91 (0.87, 0.95) * | 0.88 (0.83, 0.94) * | 0.84 (0.78, 0.90) * |
| **Preference for whole grain products** | 0.89 (0.85, 0.93) * | 0.87 (0.82, 0.93) * | 0.83 (0.77, 0.89) * |
| **Snacks** | 0.90 (0.86, 0.94) * | 0.88 (0.82, 0.94) * | 0.84 (0.78, 0.90) * |
| **Healthy beverages consumption** | 0.91 (0.87, 0.95) * | 0.87 (0.81, 0.92) * | 0.83 (0.77, 0.89) * |
| **Limited consumption of sugar-sweetened beverages** | 0.91 (0.87, 0.95) * | 0.87 (0.82, 0.93) * | 0.84 (0.78, 0.90) * |
| **Physical activity** | 0.92 (0.87, 0.96) * | 0.87 (0.82, 0.93) * | 0.84 (0.78, 0.90) * |
| **Nap** | 0.91 (0.87, 0.95) * | 0.88 (0.83, 0.94) * | 0.84 (0.79, 0.91) * |
| **Hours of sleep** | 0.88 (0.84, 0.92) * | 0.88 (0.82, 0.93) * | 0.84 (0.78, 0.90) * |
| **Limit sedentary activities** | 0.92 (0.88, 0.96) * | 0.87 (0.82, 0.93) * | 0.84 (0.78, 0.90) * |
| **Collective sports** | 0.91 (0.87, 0.95) * | 0.87 (0.81, 0.93) * | 0.83 (0.78, 0.89) * |
| **Socializing with friends or family** | 0.90 (0.86, 0.95) * | 0.87 (0.82, 0.93) * | 0.83 (0.77, 0.89) * |
| 1 vs. 0 points.  Adjusted for age, sex, education, race, and deprivation index, body mass index, smoking status, and total energy intake, duration of diabetes, diabetic medication use, macro-vascular diseases.  *, *P* < 0.05; HR, Hazard Ratios; CI, Confidence Intervals; T2D, Type 2 diabetes. | | | |

**Table S5.** The association between MEDLIFE blocks and the risk of T2D and microvascular complications in participants with prediabetes.

|  |  | **HR (95% CI)** |
| --- | --- | --- |
| **T2D** | block one | 1.00 (0.93, 1.07) |
|  | block two | 0.87 (0.79, 0.95) * |
|  | block three | 0.91 (0.83, 0.99) * |
|  |  |  |
| **Any microvascular complication** | block one | 0.89 (0.80, 0.98) * |
|  | block two | 0.90 (0.79, 1.03) |
|  | block three | 0.87 (0.77, 0.99) * |
|  |  |  |
| **Diabetic nephropathy** | block one | 0.84 (0.76, 0.94) * |
|  | block two | 0.86 (0.74, 1.00) * |
|  | block three | 0.85 (0.73, 0.98) * |
| Adjusted for age, sex, education, race, and deprivation index, smoking status, baseline cardiovascular diseases, baseline hypertension, medication use for cholesterol, blood pressure or diabetes, and total energy intake and VAT.  *, *P* < 0.05; HR, Hazard Ratios; CI, Confidence Intervals; T2D, Type 2 diabetes; VAT: visceral adipose tissue; MEDLIFE: Mediterranean lifestyle.  Block 1: Mediterranean food consumption;  Block 2: Mediterranean dietary habits;  Block 3: Physical activity, rest, social habits, and conviviality | | |

**Table S6.** The association of MEDLIFE index with risk of T2D and microvascular complications in participants with prediabetes, considering death as competing event.

|  | **Q1** | **Q2** | **Q3** | **Q4** | ***P* for trend** | **Per two points increase** |
| --- | --- | --- | --- | --- | --- | --- |
|  |  | **HR (95% CI)** | **HR (95% CI)** | **HR (95% CI)** |  | **HR (95% CI)** |
| **T2D** | ref | 1.01 (0.87, 1.17) | 0.93 (0.82, 1.06) | 0.83 (0.70, 0.97) * | 0.022 | 0.94 (0.90, 0.98) * |
| **Any microvascular complication** | ref | 0.85 (0.68, 1.05) | 0.76 (0.63, 0.92) * | 0.70 (0.56, 0.88) * | <0.001 | 0.89 (0.84, 0.95) * |
| **Diabetic nephropathy** | ref | 0.78 (0.61, 0.99) * | 0.74 (0.60, 0.90) * | 0.59 (0.46, 0.76) * | <0.001 | 0.85 (0.80, 0.91) * |
| Adjusted for age, sex, education, race, and deprivation index, body mass index, smoking status, and total energy intake, duration of diabetes, diabetic medication use.  *, *P* < 0.05; Q1, Quartile 1; Q2, Quartile 2; Q3, Quartile 3; Q4, Quartile 4; ref, reference.; HR, Hazard Ratios; CI, Confidence Intervals; T2D, Type 2 diabetes. | | | | | | |

**Table S7.** The association of VAT with risk of T2D and microvascular complications in participants with prediabetes, considering death as competing event.

|  | **Q1** | **Q2** | **Q3** | **Q4** | ***P* for trend** | **Per SD increase** |
| --- | --- | --- | --- | --- | --- | --- |
|  |  | **HR (95% CI)** | **HR (95% CI)** | **HR (95% CI)** |  | **HR (95% CI)** |
| **T2D** | ref | 1.78 (1.40, 2.26) * | 2.39 (1.83, 3.11) * | 2.49 (1.72, 3.59) * | <0.001 | 1.74 (1.64, 1.85) * |
| **Any microvascular complication** | ref | 1.01 (0.76, 1.34) | 1.19 (0.85, 1.68) | 1.49 (0.89, 2.50) | <0.001 | 1.26 (1.15, 1.38) * |
| **Diabetic nephropathy** | ref | 1.00 (0.74, 1.37) | 1.19 (0.82, 1.74) | 1.60 (1.00, 2.78) * | <0.001 | 1.23 (1.11, 1.37) * |
| Adjusted for age, sex, education, race, and deprivation index, body mass index, smoking status, and total energy intake, duration of diabetes, diabetic medication use.  *, *P* < 0.05; Q1, Quartile 1; Q2, Quartile 2; Q3, Quartile 3; Q4, Quartile 4; ref, reference.; HR, Hazard Ratios; CI, Confidence Intervals; T2D, Type 2 diabetes. | | | | | | |

**Table S8.** The association of MEDLIFE index with risk of T2D and microvascular complications in participants with prediabetes, excluding cases within first two years of follow-up.

|  | **Q1** | **Q2** | **Q3** | **Q4** | ***P* for trend** | **Per two points increase** |
| --- | --- | --- | --- | --- | --- | --- |
|  |  | **HR (95% CI)** | **HR (95% CI)** | **HR (95% CI)** |  | **HR (95% CI)** |
| **T2D** | ref | 1.03 (0.89, 1.21) | 0.94 (0.82, 1.08) | 0.82 (0.70, 0.97) * | 0.031 | 0.94 (0.90, 0.99) * |
| **Any microvascular complication** | ref | 0.84 (0.67, 1.05) | 0.76 (0.63, 0.91) * | 0.69 (0.55, 0.86) * | <0.001 | 0.88 (0.83, 0.94) * |
| **Diabetic nephropathy** | ref | 0.78 (0.61, 1.00) * | 0.74 (0.60, 0.90) * | 0.59 (0.46, 0.75) * | <0.001 | 0.85 (0.79, 0.91) * |
| Adjusted for age, sex, education, race, and deprivation index, body mass index, smoking status, and total energy intake, duration of diabetes, diabetic medication use.  *, *P* < 0.05; Q1, Quartile 1; Q2, Quartile 2; Q3, Quartile 3; Q4, Quartile 4; ref, reference.; HR, Hazard Ratios; CI, Confidence Intervals; T2D, Type 2 diabetes. | | | | | | |

**Table S9.** The association of VAT with risk of T2D and microvascular complications in participants with prediabetes, excluding cases within first two years of follow-up.

|  | **Q1** | **Q2** | **Q3** | **Q4** | ***P* for trend** | **Per SD increase** |
| --- | --- | --- | --- | --- | --- | --- |
|  |  | **HR (95% CI)** | **HR (95% CI)** | **HR (95% CI)** |  | **HR (95% CI)** |
| **T2D** | ref | 2.24 (1.77, 2.85) * | 3.84 (3.04, 4.84) * | 6.26 (4.92, 7.96) * | <0.001 | 1.78 (1.67, 1.89) * |
| **Any microvascular complication** | ref | 1.04 (0.81, 1.34) | 1.29 (1.00, 1.68) | 1.77 (1.35, 2.34) * | <0.001 | 1.26 (1.15, 1.39) * |
| **Diabetic nephropathy** | ref | 1.02 (0.77, 1.35) | 1.20 (0.90, 1.59) | 1.67 (1.24, 2.27) * | <0.001 | 1.24 (1.11, 1.37) * |
| Adjusted for age, sex, education, race, and deprivation index, body mass index, smoking status, and total energy intake, duration of diabetes, diabetic medication use.  *, *P* < 0.05; Q1, Quartile 1; Q2, Quartile 2; Q3, Quartile 3; Q4, Quartile 4; ref, reference.; HR, Hazard Ratios; CI, Confidence Intervals; T2D, Type 2 diabetes. | | | | | | |

**Table S10.** The association of MEDLIFE index with risk of T2D and microvascular complications in participants with prediabetes, among participants who completed three or more 24 hours dietary assessments.

|  | **Q1** | **Q2** | **Q3** | **Q4** | ***P* for trend** | **Per two points increase** |
| --- | --- | --- | --- | --- | --- | --- |
|  |  | **HR (95% CI)** | **HR (95% CI)** | **HR (95% CI)** |  | **HR (95% CI)** |
| **T2D** | ref | 0.97 (0.80, 1.17) | 0.94 (0.80, 1.12) | 0.94 (0.77, 1.15) | 0.449 | 0.97 (0.92, 1.02) |
| **Any microvascular complication** | ref | 0.76 (0.58, 1.00) | 0.67 (0.52, 0.86) * | 0.62 (0.46, 0.83) * | <0.001 | 0.85 (0.78, 0.92) * |
| **Diabetic nephropathy** | ref | 0.66 (0.48, 0.90) * | 0.64 (0.49, 0.84) * | 0.51 (0.36, 0.71) * | <0.001 | 0.81 (0.74, 0.88) * |
| Adjusted for age, sex, education, race, and deprivation index, body mass index, smoking status, and total energy intake, duration of diabetes, diabetic medication use.  *, *P* < 0.05; Q1, Quartile 1; Q2, Quartile 2; Q3, Quartile 3; Q4, Quartile 4; ref, reference.; HR, Hazard Ratios; CI, Confidence Intervals; T2D, Type 2 diabetes. | | | | | | |

**Table S11.** The association of VAT with risk of T2D and microvascular complications in participants with prediabetes, among participants who completed three or more 24 hours dietary assessments.

|  | **Q1** | **Q2** | **Q3** | **Q4** | ***P* for trend** | **Per SD increase** |
| --- | --- | --- | --- | --- | --- | --- |
|  |  | **HR (95% CI)** | **HR (95% CI)** | **HR (95% CI)** |  | **HR (95% CI)** |
| **T2D** | ref | 1.99 (1.50, 2.63) * | 3.22 (2.45, 4.23) * | 5.56 (4.20, 7.38) * | <0.001 | 1.78 (1.64, 1.92) * |
| **Any microvascular complication** | ref | 1.06 (0.77, 1.45) | 1.22 (0.87, 1.69) | 1.85 (1.31, 2.61) * | <0.001 | 1.29 (1.14, 1.45) * |
| **Diabetic nephropathy** | ref | 1.03 (0.72, 1.47) | 1.26 (0.87, 1.81) | 1.84 (1.25, 2.71) * | 0.001 | 1.27 (1.11, 1.46) * |
| Adjusted for age, sex, education, race, and deprivation index, body mass index, smoking status, and total energy intake, duration of diabetes, diabetic medication use.  *, *P* < 0.05; Q1, Quartile 1; Q2, Quartile 2; Q3, Quartile 3; Q4, Quartile 4; ref, reference.; HR, Hazard Ratios; CI, Confidence Intervals; T2D, Type 2 diabetes. | | | | | | |

**Table S12.** The association of MEDLIFE index with risk of T2D and microvascular complications, among subjects meeting the criteria for prediabetes as delineated by HbA1c levels.

|  | **Q1** | **Q2** | **Q3** | **Q4** | ***P* for trend** | **Per two points increase** |
| --- | --- | --- | --- | --- | --- | --- |
|  |  | **HR (95% CI)** | **HR (95% CI)** | **HR (95% CI)** |  | **HR (95% CI)** |
| **T2D** | ref | 1.01 (0.87, 1.17) | 0.93 (0.81, 1.06) | 0.83 (0.70, 0.97) * | 0.023 | 0.94 (0.90, 0.98) * |
| **Any microvascular complication** | ref | 0.85 (0.68, 1.05) | 0.76 (0.63, 0.92) * | 0.71 (0.57, 0.88) * | <0.001 | 0.89 (0.84, 0.95) * |
| **Diabetic nephropathy** | ref | 0.78 (0.61, 1.00) * | 0.74 (0.60, 0.90) * | 0.60 (0.46, 0.77) * | <0.001 | 0.85 (0.79, 0.91) * |
| Adjusted for age, sex, education, race, and deprivation index, body mass index, smoking status, and total energy intake, duration of diabetes, diabetic medication use.  *, *P* < 0.05; Q1, Quartile 1; Q2, Quartile 2; Q3, Quartile 3; Q4, Quartile 4; ref, reference.; HR, Hazard Ratios; CI, Confidence Intervals; T2D, Type 2 diabetes. | | | | | | |

**Table S13.** The association of VAT with risk of T2D and microvascular complications, among subjects meeting the criteria for prediabetes as delineated by HbA1c levels.

|  | **Q1** | **Q2** | **Q3** | **Q4** | ***P* for trend** | **Per SD increase** |
| --- | --- | --- | --- | --- | --- | --- |
|  |  | **HR (95% CI)** | **HR (95% CI)** | **HR (95% CI)** |  | **HR (95% CI)** |
| **T2D** | ref | 2.16 (1.72, 2.72) * | 3.66 (2.92, 4.58) * | 5.87 (4.66, 7.41) * | <0.001 | 1.75 (1.65, 1.86) * |
| **Any microvascular complication** | ref | 1.03 (0.80, 1.33) | 1.28 (0.99, 1.65) | 1.80 (1.37, 2.37) * | <0.001 | 1.27 (1.16, 1.40) * |
| **Diabetic nephropathy** | ref | 0.99 (0.75, 1.31) | 1.19 (0.89, 1.58) | 1.70 (1.26, 2.31) * | <0.001 | 1.24 (1.12, 1.38) * |
| Adjusted for age, sex, education, race, and deprivation index, body mass index, smoking status, and total energy intake, duration of diabetes, diabetic medication use.  *, *P* < 0.05; Q1, Quartile 1; Q2, Quartile 2; Q3, Quartile 3; Q4, Quartile 4; ref, reference.; HR, Hazard Ratios; CI, Confidence Intervals; T2D, Type 2 diabetes. | | | | | | |

**
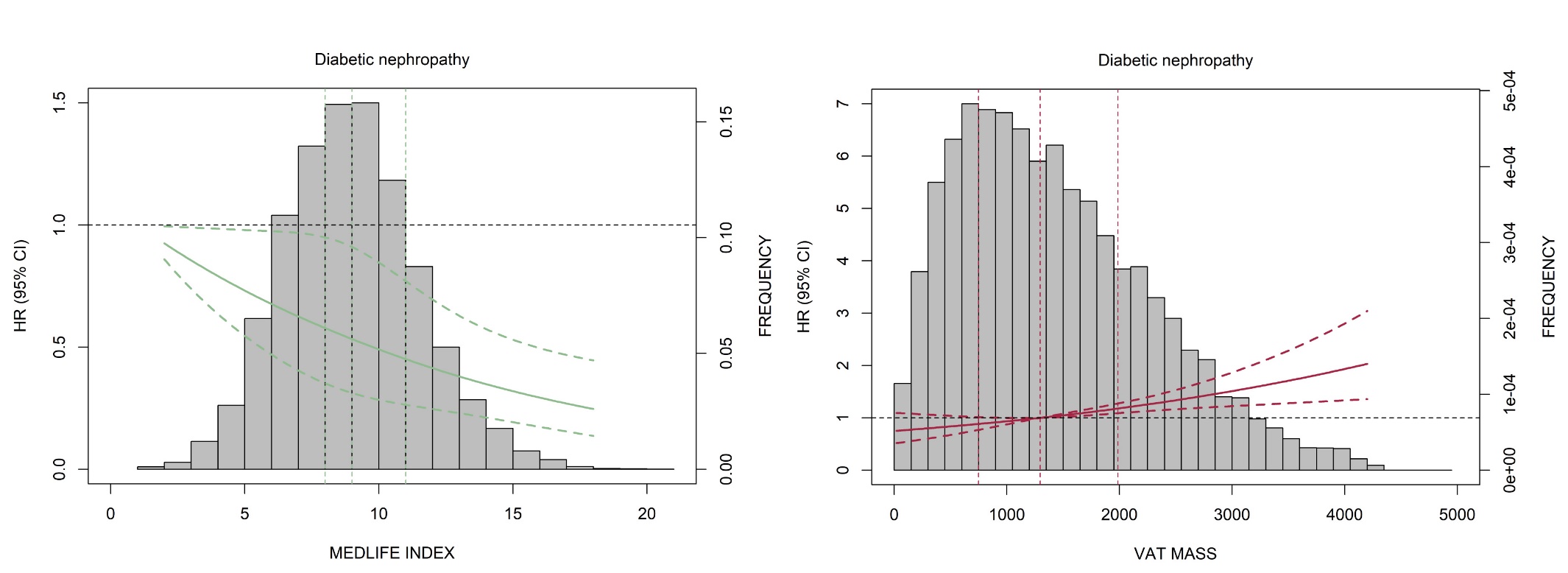
Fig S1. Association between MEDLIFE INDEX and VAT with diabetic nephropathy.** Plotted values are HR (95% CI) from a restricted cubic spline Cox regression model, with a MEDLIFE INDEX of 1 point and VAT mass of 1300 gram (the median) as references. The vertical dashed line represents the quartile line. Adjusted for age, sex, education, race, and deprivation index, smoking status, baseline cardiovascular diseases, baseline hypertension, medication use for cholesterol, blood pressure or diabetes, and total energy intake, and MEDLIFE score or VAT. HR, Hazard Ratios; CI, Confidence Intervals; T2D, Type 2 diabetes; VAT: visceral adipose tissue; MEDLIFE: Mediterranean lifestyle.

**
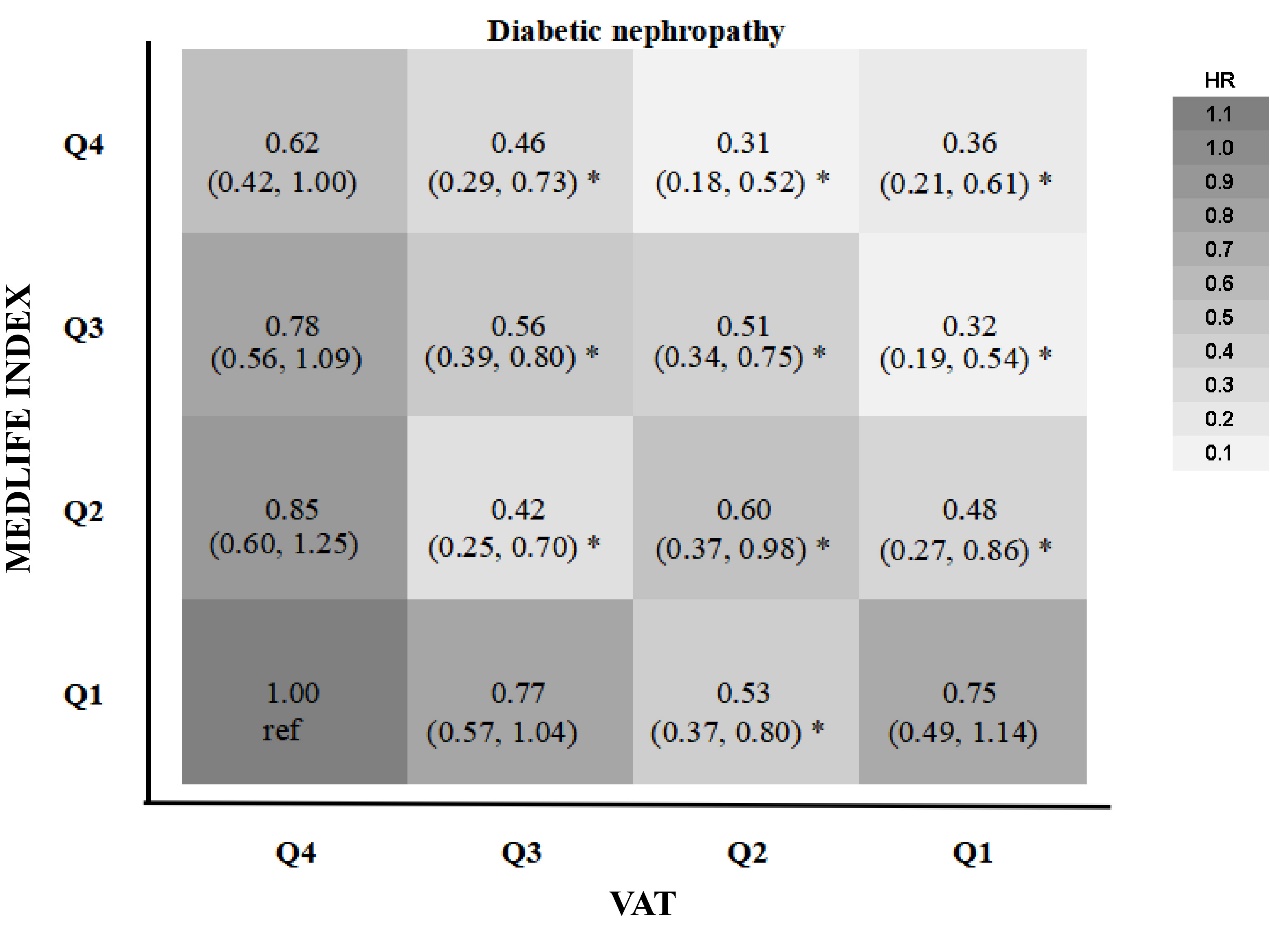
**

**Fig. S2 Associations of combinations of MEDLIFEN Index and VAT mass with incident diabetic nephropathy.** Estimated by multivariable-adjusted HRs by use of Cox regression analysis with the highest category of VAT and in the lowest category of MEDLIFE as the reference. Adjusted for age, sex, education, race, and deprivation index, smoking status, baseline cardiovascular diseases, baseline hypertension, medication use for cholesterol, blood pressure or diabetes, and total energy intake. Ref, reference.; HR, Hazard Ratios; CI, Confidence Intervals; T2D, Type 2 diabetes; VAT: visceral adipose tissue; MEDLIFE: Mediterranean lifestyle
